# Mycoviruses: Past, Present, and Future

**DOI:** 10.3390/v11040361

**Published:** 2019-04-19

**Authors:** Ioly Kotta-Loizou

**Affiliations:** Department of Life Sciences, Imperial College London, London SW7 2AZ, UK; i.kotta-loizou13@imperial.ac.uk

Approximately a year ago, when I accepted the offer to act as a Guest Editor for the Special Issue ‘Mycoviruses’ organised by the MDPI journal *Viruses*, I dared not expect that ‘Mycoviruses’ would include such a large number of manuscripts. Therefore, it is with great delight that I can count today a total of twenty-three, high-quality publications and I would like to take this opportunity to thank deeply all the contributing authors who chose ‘Mycoviruses’ as a vehicle for sharing their fascinating work with the mycovirology community.

‘Mycoviruses’ consists of three timely reviews on virus structure [1] and viruses of significant fungal pathogens causing damage to forests [2] and crops [3], together with twenty original research articles covering a range of relevant topics. These include the discovery and characterisation of novel members in the families *Chrysoviridae*, *Endornaviridae*, *Hypoviridae*, *Mymonaviridae*, *Narnaviridae*, *Partitiviridae*, *Totiviridae*, *Quadriviridae,* and others [4,5,6,7,8,9,10,11,12,13,14,15,16]; the study of the distribution, transmission, evolution, and dynamics of viruses in fungal populations [5,9,10,14,16,17,18] and the development of new techniques and methods to be used in the field of mycovirology [19,20]. Of particular interest are the investigations regarding the effects of viruses on their fungal hosts, most prominently on fungal morphology, spore production, growth, virulence [4,8,9,13,14,15,17,21,22,23], and, in the case of killer yeast systems, toxin production [6,20]. Understanding how these effects are mediated is crucial and applications of high-throughput next-generation sequencing technologies such as transcriptome and small RNA profiling provide insight into the molecular mechanisms underpinning the observed phenotypes [21,22], in addition to increasing sensitivity of virus detection [12,15,16]. Furthermore, the link between antiviral RNA silencing and infection is undoubtedly a significant one, given its implications for virus-induced hypovirulence, hypervirulence, and other phenotypic alterations [22,23]. 

As a relatively young mycovirologist with hopefully many active years to look forward to and a scientist with some first-hand knowledge in animal and plant virology, I frequently question how our work on mycoviruses conforms with the rapidly developing research in other fields and which future directions will lead to mycovirology being rightfully accepted as a mainstream research area and not merely esoteric, as it is often regarded. Mycovirology is not as advanced as human, animal, or even plant virology; overall our understanding of mycoviruses is not as detailed and in depth while the methodology we use to study them is not at the cutting edge. Since mycoviruses are not causative agents of significant diseases, they receive much less attention than, for instance, life-threatening human pathogens. Their ‘moment of glory’ was the use of *Cryphonectria parasitica* hypoviruses to control chestnut blight in Europe during the last century and today the major interest stems from the potential of mycoviruses as biological control agents in the context of integrative pest management programs. Based on this observation, a way forward would be to genetically engineer mycovirus-mediated hypovirulence or hypervirulence instead of merely hoping for a fortunate discovery. To this end, both the understanding of the molecular mechanisms underpinning these phenotypes and the development of reverse genetics systems for mycoviruses is an essential prerequisite.

## References

[1] Luque D., Mata C.P., Suzuki N., Ghabrial S.A., Castón J.R. (2018). Capsid structure of dsRNA fungal viruses. Viruses.

[2] Botella L., Hantula J. (2018). Description, distribution, and relevance of viruses of the forest pathogen *Gremmeniella abietina*. Viruses.

[3] Moriyama H., Urayama S.I., Higashiura T., Le T.M., Komatsu K. (2018). Chrysoviruses in *Magnaporthe oryzae*. Viruses.

[4] Hao F., Ding T., Wu M., Zhang J., Yang L., Chen W., Li G. (2018). Two novel hypovirulence-associated mycoviruses in the phytopathogenic fungus *Botrytis cinerea*: Molecular characterization and suppression of infection cushion formation. Viruses.

[5] Hao F., Wu M., Li G. (2018). Molecular characterization and geographic distribution of a mymonavirus in the population of *Botrytis cinerea*. Viruses.

[6] Vepštaitė-Monstavičė I., Lukša J., Konovalovas A., Ežerskytė D., Stanevičienė R., Strazdaitė-Žielienė Ž., Serva S., Servienė E. (2018). *Saccharomyces paradoxus* K66 killer system evidences expanded assortment of helper and satellite viruses. Viruses.

[7] Chun J., Yang H.E., Kim D.H. (2018). Identification and molecular characterization of a novel partitivirus from *Trichoderma atroviride* NFCF394. Viruses.

[8] Mizutani Y., Abraham A., Uesaka K., Kondo H., Suga H., Suzuki N., Chiba S. (2018). Novel mitoviruses and a unique tymo-like virus in hypovirulent and virulent strains of the Fusarium head blight fungus, *Fusarium boothii*. Viruses.

[9] Yang D., Wu M., Zhang J., Chen W., Li G., Yang L. (2018). Sclerotinia minor endornavirus 1, a novel pathogenicity debilitation-associated mycovirus with a wide spectrum of horizontal transmissibility. Viruses.

[10] Schoebel C.N., Prospero S., Gross A., Rigling D. (2018). Detection of a conspecific mycovirus in two closely related native and introduced fungal hosts and evidence for interspecific virus transmission. Viruses.

[11] Liu C., Zeng M., Zhang M., Shu C., Zhou E. (2018). Complete nucleotide sequence of a partitivirus from *Rhizoctonia solani* AG-1 IA strain C24. Viruses.

[12] Neupane A., Feng C., Feng J., Kafle A., Bücking H., Lee Marzano S.Y. (2018). Metatranscriptomic analysis and *in silico* approach identified mycoviruses in the arbuscular mycorrhizal fungus *Rhizophagus* spp.. Viruses.

[13] Shah U.A., Kotta-Loizou I., Fitt B.D.L., Coutts R.H.A. (2019). Identification, molecular characterization, and biology of a novel quadrivirus infecting the phytopathogenic fungus *Leptosphaeria biglobosa*. Viruses.

[14] Kamaruzzaman M., He G., Wu M., Zhang J., Yang L., Chen W., Li G. (2019). A novel partitivirus in the hypovirulent isolate QT5-19 of the plant pathogenic fungus *Botrytis cinerea*. Viruses.

[15] Tran T.T., Li H., Nguyen D.Q., Jones M.G.K., Wylie S.J. (2019). Co-infection with three mycoviruses stimulates growth of a *Monilinia fructicola* isolate on nutrient medium, but does not induce hypervirulence in a natural host. Viruses.

[16] Nibert M.L., Debat H.J., Manny A.R., Grigoriev I.V., De Fine Licht H.H. (2019). Mitovirus and mitochondrial coding sequences from basal fungus *Entomophthora muscae*. Viruses.

[17] Filippou C., Garrido-Jurado I., Meyling N.V., Quesada-Moraga E., Coutts R.H.A., Kotta-Loizou I. (2018). Mycoviral population dynamics in Spanish isolates of the entomopathogenic fungus *Beauveria bassiana*. Viruses.

[18] Rigling D., Borst N., Cornejo C., Supatashvili A., Prospero S. (2018). Genetic and phenotypic characterization of Cryphonectria hypovirus 1 from Eurasian Georgia. Viruses.

[19] Özkan-Kotiloğlu S., Coutts R.H.A. (2018). Multiplex detection of *Aspergillus fumigatus* mycoviruses. Viruses.

[20] Crabtree A.M., Kizer E.A., Hunter S.S., Van Leuven J.T., New D.D., Fagnan M.W., Rowley P.A. (2019). A rapid method for sequencing double-stranded RNAs purified from yeasts and the identification of a potent K1 killer toxin isolated from *Saccharomyces cerevisiae*. Viruses.

[21] Ejmal M.A., Holland D.J., MacDiarmid R.M., Pearson M.N. (2018). The effect of Aspergillus thermomutatus chrysovirus 1 on the biology of three *Aspergillus* species. Viruses.

[22] Lee Marzano S.Y., Neupane A., Domier L. (2018). Transcriptional and small RNA responses of the white mold fungus *Sclerotinia sclerotiorum* to infection by a virulence-attenuating hypovirus. Viruses.

[23] Mochama P., Jadhav P., Neupane A., Lee Marzano S.Y. (2018). Mycoviruses as triggers and targets of RNA silencing in white mold fungus *Sclerotinia sclerotiorum*. Viruses.

